# Circumstances surrounding dying in the paediatric intensive care unit

**DOI:** 10.1186/1471-2431-6-22

**Published:** 2006-08-07

**Authors:** Jetske ten Berge, Dana-Anne H de Gast-Bakker, Frans B Plötz

**Affiliations:** 1 BSc, Department of Paediatric Intensive Care, VU Medical Center, Amsterdam, The Netherlands; 2 Paediatrician, fellow paediatric intensive care, Department of Paediatric Intensive Care, VU Medical Center, Amsterdam, The Netherlands; 3 Paediatric intensivist, Department of Paediatric Intensive Care, VU Medical Center, Amsterdam, The Netherlands

## Abstract

**Background:**

Death is inevitable in the paediatric intensive care unit (PICU). We aimed to describe the circumstances surrounding dying in a PICU.

**Method:**

The chart records of all patients less than 18 years of age who died at the PICU between January first 2000 and July first 2005 were retrospectively analyzed. Information regarding sex, age, length of stay, admission, diagnosis, and the way a patient died was registered. Post mortem information regarding natural versus unnatural death, autopsy and donation was obtained. Non-survivors were allocated in five groups: do-not-resuscitate (DNR), withholding and/or withdrawal of therapy (W/W), failed cardiopulmonary resuscitation (failed CPR), brain death (BD), and terminal organ failure (TOF).

**Results:**

During the study period 87 (4.4%) of the 1995 admitted patients died. Non-survivors were more often admitted during the day (54%) and the week (68%). W/W was found in 27.6%, TOF in 26.4%, BD in 23.0%, failed CPR in 18.4%, and DNR in 4.6%. Forty-three percent died in the first two days, of which BD (40.5%) and failed CPR (37.8%) were most common. Seventy-five children (86%) died due to a natural cause. Autopsy permission was obtained in 19 of 54 patients (35%). The autopsies confirmed the clinical diagnosis in 11 patients, revealed new information in 5 patients, and in 3 patients the autopsy did not provide additional information. Nine patients were medically suitable for organ donation and 24 patients for tissue donation, whereas consent was only obtained in 2 cases in both groups.

**Conclusion:**

We observed that 43% of the patients died within the first two days of admission due to BD and failed CPR, whereas after 4 days most patients died after W/W. Autopsy remains an useful tool to confirm clinical diagnoses or to provide new information. Only a small percentage of the deceased children is suitable for organ donation.

## Background

Death is inevitable in the paediatric intensive care unit (PICU). Mortality rates differ from 3,8% to 13% in PICUs in North and South America and Europe [1-4]. Nowadays practice of withholding and withdrawal of treatment (W/W) and do not resuscitate (DNR) orders in children are medically and ethically acceptable under certain circumstances. In North America and Europe 28% to 65% of all PICU deaths follow after a restriction in care [5-8]. On the other hand, studies performed at adult ICUs indicate that mortality rates are affected by several factors, such as time, day, and source of admission [9,10]. However, whether this holds true for PICUs is unclear due to lack of studies addressing these factors.

The present study was therefore undertaken to describe circumstances surrounding dying in the PICU. In addition, post mortem procedures like autopsy and donation in this group of paediatric patients are described.

## Methods

### Patients

Our PICU is a 9-bed combined medical and surgical (excluding cardiac surgery) intensive care unit, staffed by trained paediatric intensivists, located in a university hospital. The chart records of all patients less than 18 years of age who died at the PICU between January first 2000 and July first 2005 were retrospectively analyzed. For a retrospective review of this nature it is not institutional policy to require ethical committee approval.

### Demographic data

Information regarding sex, age, length of stay, time and day of admission were recorded. The time of admission was classified as daytime (between 8 AM and 5 PM), evening (between 5 PM and 11 PM), or night (between 11 PM and 8 AM). The day of admission was classified as weekday or weekend day (between Friday 5 PM and Monday 8 AM).

The reason for admission was categorized as follows: (a) acute congenital disease, present at birth or later in life, (b) acute acquired disease, not related to any chronic condition, (c) complication/exacerbation or natural progression of a chronic disease, (d) chronic congenital disease, (e) chronic acquired disease, and (f) post operative care following elective surgery. Chronic disease status was defined as a condition persisting for more than 30 days.

The admission diagnosis was categorized as follows: (a) respiratory, (b) circulatory, (c) neurological, (d) metabolic/internal environment, (e) trauma and (f) other, including post cardiopulmonary resuscitation and post operative care. The source of admission was categorized as: (a) emergency room (ER), patients who were admitted after ER or family doctor visit or after emergency surgery were placed in this category, (b) post operative, following elective surgery, (c) other hospital, including ER, ward and PICU of that hospital and (d) paediatric ward of own hospital.

### Mode of death

The mode of death was allocated to five groups; (a) brain death (BD), BD was defined according to the Dutch criteria [11], (b) failed cardiopulmonary resuscitation (failed CPR), (c) do not resuscitate (DNR) (d) withholding and/or withdrawal of therapy (W/W), and (e) terminal organ failure (TOF), defined as children who were dying despite maximal treatment due to terminal organ failure. In the TOF group no children were included with failed CPR. The time of death concerning brain death patients was determined as the time when the diagnosis brain death was confirmed [11]. In case patients underwent organ donation death was declared after their last isoelectric electroencephalogram.

### Post mortem

In the Netherlands, the attending physician determines whether the patient died due to a natural or an unnatural cause [12]. Unnatural death is defined as death by external cause, such as drowning, suffocation or violence, whether intentional or not. When the attending physician concludes that there is an unnatural death, or if the physician has doubts about the cause of death, the district medical examiner is called. This examiner determines the cause of death and whether an forensic autopsy is required or whether the body is released. Donation, in case of an unnatural death, is possible when the body is released or after the forensic autopsy.

On autopsy the following information was registered: first whether the doctors approached the family for obtaining consent for autopsy, second whether consent was obtained and third whether the autopsy provided additional information. An autopsy was considered to provide additional information when the diagnosis before death was confirmed or when the autopsy revealed new information.

On donation we registered if patients were medically suitable for organ and/or tissue donation, whether families were approached for consent, whether consent was obtained and whether the donation actually took place. The criteria to determine whether the patient was medically suitable for donation were acquired from the Dutch transplantation foundation. For organ donation brain death is required including strict criteria to confirm the diagnosis brain death [12]. Parents of patients younger than 1 year old were, according to the transplantation protocol, not actively approached for tissue donation.

### Statistical analysis

Differences of categorical variables among the modes of death (such as reason of admission and admission diagnose) were investigated by the chi-square test or the Fisher exact test. The measured variables of age and length of stay were, due to non-normality distributions, analyzed by non-parametric methods (Kruskal-Wallis test and Mann-Whitney test) for the comparison among the modes of death. A p value < 0.05 was considered to be statistically significant.

## Results

### Patients

During the study period 1995 patients were admitted at the PICU, of whom 87 (4.4%) died (table 1). About two-third (67.8%) of the non-survivors were admitted during the week. Most patients (54.0%) were admitted during the day, whereas 18% and 28% were admitted during evening or at night, respectively. About 39% of the non-survivors were admitted from another hospital (table 1). Of the inpatients admitted at the PICU (61%), 28% was referred from the ER, 23% from the paediatric ward and 10% from the operating room. Patients admitted from the ER were older (median 68.4 months, p < 0.05) whereas patients admitted from another hospital were younger (median 13.7 months, p < 0.05). Admission from the ER or another hospital was followed by a shorter length of stay (both medians 2 days, p < 0.01 and p < 0.05, respectively). Admission from the paediatric ward of our own hospital resulted in a longer length of stay (median 16,5 days, p < 0.01).

**Table 1:** Clinical characteristics and mode of death of the non-survivors. Differences of the measured variables age and length of stay and differences of the categorical variables among the modes of death (* p < 0.05, **p < 0.01). (*DNR *do not resuscitate, *W/W *withholding or withdrawal of therapy, *failed CPR *failed cardiopulmonary resuscitation, *BD *brain death, *TOF *terminal organ failure, *exa/compl chron disease *exacerbation/complication of a chronic disease)

	**Total**	**DNR**	**W/W**	**failed CPR**	**BD**	**TOF**
	(n = 87)	(n = 4)	(n = 24)	(n = 16)	(n = 20)	(n = 23)
Male patients (%)	49 (56.3)	2 (50.0)	13 (54.2)	9 (56.3)	11(55.0)	14 (60.9)
Median age (months)	26.4	125.8	53.1	10.9**	32.4	48.7
Median length of stay(days)	4.0	7.5	7.5**	2.0	2.0**	5.5
						
**Reasons of admission (%)**						
Acute congenital disease	9 (10.3)		1 (4.2)	2 (12.5)	4 (20.0)	2 (8.7)
Acute acquired disease	56 (64.5)	2 (50.0)	14 (58.3)	11 (68.7)	15 (75)	14 (60.9)
Exa/compl chron disease	13 (14.9)	2 (50.0)	5 (20.8)*		1 (5.0)	5 (21.7)*
Post operative care	9 (10.3)		4 (16.7)	3 (18.8)		2 (8.7)
						
**Admission diagnosis (%)**						
Respiratory	21 (24.2)	2 (50.0)	8 (33.3)	5 (31.3)	2 (10.0)	4 (17.4)
Circulatory	16 (18.4)		2 (8.4)	4 (25.0)	1 (5.0)	9 (39.2)*
Neurological	22 (25.3)	2 (50.0)	6 (25.0)	2 (12.4)	10(50.0)*	2 (8.7)
Metabolic/Internal environment	3 (3.4)					3 (13.0)
Trauma	2 (2.3)				1 (5.0)	1 (4.3)
Other	23 (26.4)		8 (33.3)	5 (31.3)	6 (30.0)	4 (17.4)
						
**Source (%)**						
ER	24 (27.6)	3 (75)	5 (20.8)	3 (18.8)	10 (50.0)*	3 (13.0)
Post operative	9 (10.3)		4 (16.7)	3 (18.8)		2 (8.8)
Other hospital	34 (39.1)		7 (29.2)	8 (50.0)	10 (50.0)	9 (39.1)
Paediatric ward	20 (23.0)	1 (25)	8 (33.3)	2 (12.4)		9 (39.1)*

### Mode of death

Modes of death were BD in 20 (23.0%), failed CPR in 16 (18.4%), DNR in 4 (4.6%), W/W in 24 (27.6%) and TOF in 23 (26.4%) patients, respectively. Forty-three percent of the deaths occurred in the first two days, of which BD (40.5%) and failed CPR (37.8%) were most common. The percentage of deaths increased to 59% within four days. Death followed by a restriction in therapy increased after four days, whereas BD was not diagnosed anymore and failed CPR was less common. Patients in the failed CPR group were significantly younger than patients in the other groups (p < 0.01). Patients admitted with an exacerbation/complication of a chronic disease were more likely to die due to W/W or TOF (p < 0.05). BD patients more often died due to an unnatural cause (p < 0.05) and were more often admitted from the ER (p < 0.05). TOF was more common when admitted from the ward (p < 0.05).

### Post mortem

#### Natural vs unnatural cause of death

Seventy-five children (86.2%) died due to a natural cause. Fifty-four families were asked permission for autopsy, which was obtained in 19 patients (35.2%). The autopsies confirmed the clinical diagnosis in 11 patients (57.8%), whereas in 3 patients (15.8) the autopsy did not provide additional information and the cause of death remained unknown. In 5 patients (26.4%) the autopsy revealed new information about the cause of death. In one patient the intial clinical diagnosis of aspiration pneumonia was changed into bronchopneumonia and in the remaining four patients the cause of death was initially unknown. The autopsy revealed that a bronchopneumonia, a pulmonary embolism, a encephalopathy due to lymphomatoid granulomatosis, and a cerebral aneurysm respectively, as the cause of death.

Twelve children (13.8%) died due to an unnatural cause. In 3 patients this resulted in a forensic autopsy, whereas in 1 patient it was unknown whether autopsy was performed. The outcome of the forensic was not known because these autopsies are performed at the Laboratory for Forensic Pathology in Rijswijk, The Netherlands were it is not common to send a copy of the autopsy report to the attending physician.

#### Donation

In our study period (5.5 years), nine patients were medically suitable for organ donation. Six families were approached for consent and two agreed for organ donation. Both children were classified as death due to a natural cause. There were 24 patients medically suitable for tissue donation. Since ten of the 24 patients were younger than 1 year, these families were not actively approached. From the remaining 14 patients only two families were asked for consent, which was obtained in both cases. Both tissue donations were obtained from children who died due to an unnatural cause.

## Discussion

The overall mortality rate at our PICU is comparable with mortality rates reported at PICU's in North and South America and Europe [1-4]. We observed that 43% of the patients died within the first two days of admission, mainly due to BD and failed CPR. These patients were younger and were admitted primarily form the ER or from another hospital. The percentage of deaths increased to 59% within four days. After 4 days most patients died after W/W. The length of PICU stay in the W/W group was significant longer which can be explained by several factors. First, a longer period of observation is required to determine that further care is futile [1,2,4]. Second, W/W is more common after trials of therapeutic interventions, and third, it takes time to communicate with the family [4,13]. In contrast to patients by whom care was withheld or withdrawled, BD patients stayed significantly shorter in the PICU [1-5]. The incidence of DNR (4.6%) in our study was lower compared to other studies (14–27%) [2,4,5]. The meaning of a DNR order is not always the same and furthermore a DNR order is usual the first step in a process of W/W [2,5]. Limitations in therapy seem to be easier for a family then actual withdrawal of therapy [1,2]. The extra time, received through this process, could allow the family to accept their child's inevitable death. However, it could also make the situation more difficult for the family and the physicians if the clinical condition of the patient becomes more complicated. Therefore, waiting and watching is not always appropriate [2].

In the present study we defined withdrawing and withholding medical treatment as removal of life sustaining therapy or as refraining from life sustaining therapy. In contrast to Esteban et al. we did not consider DNR order in the category of withholding life support [14]. Resuscitation included acute intervention by intubation and ventilation (if not already instituted), chest compressions and/or bolus dose(s) of catecholamines and/or defibrillation to restore adequate heart rhythm, blood pressure, oxygenation and ventilation [5]. Most often therapy is withdrawn and the most common therapy withdrawn is mechanical ventilation, followed by vasopressors [15,16]. Although the decision-making process of withdrawal occurs over a relatively long period of time, after the decision was made the withdrawal process usually quicly proceeds. In general, most withdrawals are planned and occured at a prearranged time that was suitable for the family [16].

We did not observe that admission during the evening or weekend was associated with an increased mortality rate. However, this is a descriptive study and not aimed at reporting factors related to the risk of death. In addition, the small number of patients and the fact that also the total number of admissions for each day of the week is lacking, warrants to draw any firm conclusions. Arias et al observed an increased risk of death for a subgroup of patients (patients with shock, congenital cardiovascular disease or after cardiac arrest) who also revealed higher odds of death admitted to the PICU during evening hours [17]. They observed no association between mortality and the day of admission. Hixson et al reported that neither day nor time of PICU admission had a significant impact on mortality [18]. They contributed these results to the fact that the PICU was staffed 24 hrs/day, 7-days/wk by in-house, board certified intensivists. For all studies, it remains important to determine whether this observation results from difference in the structure of care, processes of care or both.

Our autopsy rate of 35 % (19/54) of those asked is comparable with other PICU's [19-21]. The autopsy rate in paediatric patients has not changed over the years despite major diagnostic developments in medicine. In our study the autopsies confirmed the clinical diagnosis or provided new information in 84.2%. This emphasizes again the importance of autopsies in general [19-23]. Through careful explanation of the advantages of an autopsy to a family, the consent rate could increase [21,24]. Furthermore education and discussion between physicians and pathologists may increase the autopsy rate [19,20,23,24].

The finding that families of paediatric patients are more willing to give consent for donation can not be confirmed with this study [25,26]. Tsai et al reported that of 199 children who fulfilled the criteria for brain death, 153 were medically suitable for organ donation [25]. Consent was obtained in 63% (81/128) of those asked. Morris et al even reported that all 18 of the families who were asked agreed to donate organs [26]. In contrast, we noted that 9 of the 20 BD patients were medically suitable and consent was obtained in 33% (2/6). A difficult physician-family relation and cultural or religious misunderstandings could be reasons for not approaching a family [25]. Also decoupling, hence the temporary separation of the discussion of brain death from the discussion of organ donation, may be an important tool to increase the consent rate [25].

Finally, we are aware that our study has several limitations. First, it is retrospective study, which may cause recall and interpretation bias and could lead to incomplete data. Second, we did not examine the discussion between the physicians and the families about the end-of-life care. We acknowledge that this is an important issue because qualitative research is extremely appropriate in the end-of-life care. The present study was not designed as qualitative study regarding the process of treatment limitation that might lead to death. Furthermore, these processes were poorly documented in the chart records and we thought that it was inappropriate to retrospectively interview families about the decision process. This important issue needs to be addressed in a prospective study. Third, the number of patients who died on the ward or at home after discharge with terminal disease and the patients who survived in spite of a DNR order or W/W are unknown. In a previous study it was found that of all in hospital pediatric deaths 71% died at the PICU [5]. Most patients dying on the ward had restrictions of care although it remained unclear if all these patients were initially discharged from the PICU [5]. Therefore, this study points out factors that could possible influence mortality but this needs to be confirmed in a prospective (muliticenter) study.

## Conclusion

We observed that 43% of the patients died within the first two days of admission due to BD and failed CPR, whereas after 4 days most patients died after W/W.

Autopsy remains an useful tool to either confirm the clinical diagnosis or to provide new information. We observed that only a small percentage of the deceased children is suitable for organ donation.

## Competing interests

The author(s) declare that they have no competing interests.

## Authors' contributions

JB conducted the study, analyzed the results, and drafted the manuscript. DGB assisted in designing the study and participated in interpreting the results and drafted the manuscript. FBP designed the study and participated in interpreting the results and drafted the manuscript. All authors read and approved the final manuscript.

## Pre-publication history

The pre-publication history for this paper can be accessed here:


